# Dual effects of radiotherapy on tumor microenvironment and its contribution towards the development of resistance to immunotherapy in gastrointestinal and thoracic cancers

**DOI:** 10.3389/fcell.2023.1266537

**Published:** 2023-10-03

**Authors:** Deyao Zhao, Yingyi Mo, Margarita E. Neganova, Yulia Aleksandrova, Edmund Tse, Vladimir N. Chubarev, Ruitai Fan, Olga A. Sukocheva, Junqi Liu

**Affiliations:** ^1^ Department of Radiation Oncology, The First Affiliated Hospital of Zhengzhou University, Zhengzhou, China; ^2^ Arbuzov Institute of Organic and Physical Chemistry, FRC Kazan Scientific Center, Russian Academy of Sciences, Kazan, Russia; ^3^ Institute of Physiologically Active Compounds at Federal Research Center of Problems of Chemical Physics and Medicinal Chemistry, Russian Academy of Sciences, Chernogolovka, Russia; ^4^ Department of Hepatology, Royal Adelaide Hospital, CALHN, Adelaide, SA, Australia; ^5^ Sechenov First Moscow State Medical University, Sechenov University, Moscow, Russia

**Keywords:** drug resistance, radiotherapy, tumor microenvironment, angiogenesis, cancer-associated fibroblasts, exosome, anti-cancer immunity

## Abstract

Successful clinical methods for tumor elimination include a combination of surgical resection, radiotherapy, and chemotherapy. Radiotherapy is one of the crucial components of the cancer treatment regimens which allow to extend patient life expectancy. Current cutting-edge radiotherapy research is focused on the identification of methods that should increase cancer cell sensitivity to radiation and activate anti-cancer immunity mechanisms. Radiation treatment activates various cells of the tumor microenvironment (TME) and impacts tumor growth, angiogenesis, and anti-cancer immunity. Radiotherapy was shown to regulate signaling and anti-cancer functions of various TME immune and vasculature cell components, including tumor-associated macrophages, dendritic cells, endothelial cells, cancer-associated fibroblasts (CAFs), natural killers, and other T cell subsets. Dual effects of radiation, including metastasis-promoting effects and activation of oxidative stress, have been detected, suggesting that radiotherapy triggers heterogeneous targets. In this review, we critically discuss the activation of TME and angiogenesis during radiotherapy which is used to strengthen the effects of novel immunotherapy. Intracellular, genetic, and epigenetic mechanisms of signaling and clinical manipulations of immune responses and oxidative stress by radiotherapy are accented. Current findings indicate that radiotherapy should be considered as a supporting instrument for immunotherapy to limit the cancer-promoting effects of TME. To increase cancer-free survival rates, it is recommended to combine personalized radiation therapy methods with TME-targeting drugs, including immune checkpoint inhibitors.

## 1 Introduction

**FIGURE 1:**
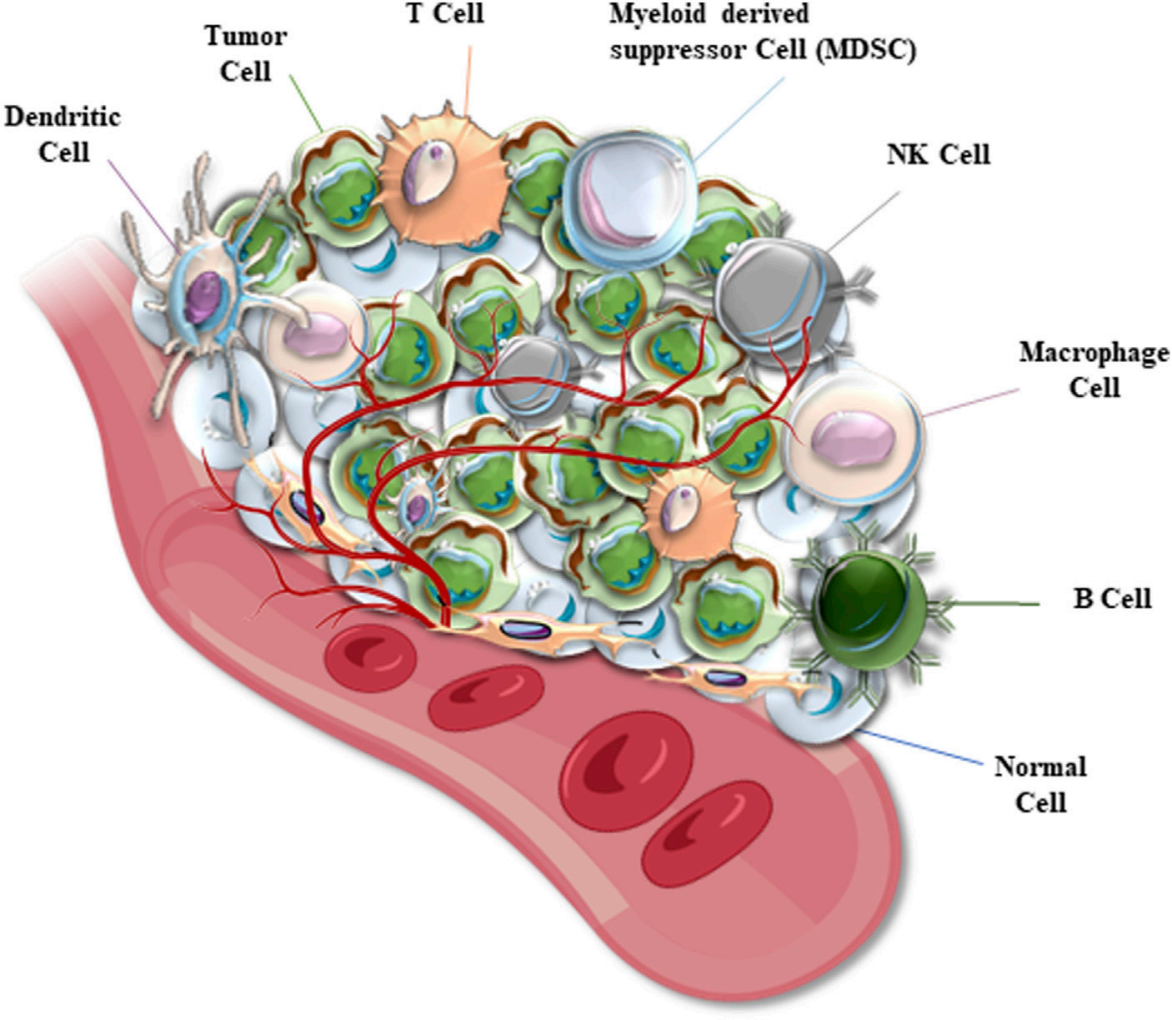
A diagram represents the structure of a solid tumor microenvironment (TME). Cancer cells are mixed with TME cells which include normal tissue cells, different immune cells, and microvessels. TEM immune cells are represented by dendritic cells (DCs), macrophages, natural killer (NK) and other T cells, B cells, myeloid-derived suppressor (MDS) cells, and others (depending on the cancer type).

The tumor microenvironment (TME) is a complex, dynamic, and cancer-orchestrated system of cells and cell-secreted components, including heterogeneous tumor cells and cancer stem cells (CSCs), cancer-associated fibroblasts (CAFs), various subsets of pro-inflammatory immune effectors, tumor-associated macrophages (TAM), endothelial components of blood and lymphatic vasculature, extracellular matrix and numerous cytokines and chemokines (Figure 1) (Liotta and Kohn, 2001; Lu et al., 2022; Xie and Su, 2022; Zhang et al., 2023a). TME is often subdivided into at least two major categories, such as a non-immune microenvironment (dominated by fibroblasts) and an immune microenvironment (dominated by immune T cells and TAM). Tumor cells and TME represent a functionally and highly dynamic structure, where tumor cells are regarded as “seeds” submerged in cancer-promoting and supporting TME (“soil”) modified during cancer immunoediting (Talbot et al., 2012; Cao et al., 2022; Lu et al., 2022; Xie and Su, 2022). According to the proposed model, tumor cells mutually interact with components of TME which is shaped to promote tumorigenesis and metastasis (Weber and Kuo, 2012).

In 2020, the number of cancer patients worldwide reached nearly twenty million, of which Asia accounted for nearly half of all cases (9,503,710 cases; 49.3%). The number of cancer cases also highly increased in China, reaching 4,568,754, compared to 2018 statistical data (Bray et al., 2018; Sung et al., 2021). The analysis of cancer therapy worldwide indicated that almost half of cancer patients received radiation treatment (Bernier, 2016; Zakeri et al., 2018). Radiotherapy is designed to kill tumor cells using high-energy ionizing radiation, such as gamma rays and X-rays, and electron and proton beams. Certain radiation doses are calculated to deliver the most efficient killing of tumor cells while sparing the normal surrounding tissues (Bernier et al., 2004; Glicksman, 2006). In sensitized cancers, radiotherapy delivers a highly desirable elimination of cancer cells, although the cancer-free outcome is hard to reach. During radiotherapy treatment, TME impacts the successful elimination of tumor cells and may negatively contribute the treatment and patient survival (Zakeri et al., 2018; Pontoriero et al., 2023). Research data confirmed that radiotherapy triggers various anti-cancer changes in TME and affects anti-tumor responses. The favorable effects of radiotherapy deliver a rationale for the combined therapeutic strategy. Application of complex treatment methods, including the use of radiotherapy as an activator of internal anti-cancer mechanisms in support of chemotherapy and immunotherapy, is recommended (Zhang et al., 2023b; Pontoriero et al., 2023; Shannon et al., 2023; Viswanath et al., 2023). In this study, we discuss the role of radiotherapy in the regulation of TME-related cancer-promoting effects. The success of targeted anti-cancer immunotherapy, combined with radiotherapy, is also discussed.

## 2 Radiation therapy effects in tumor vasculature

### 2.1 An unexpected turn: radiotherapy may promote angiogenesis and metastasis

High-energy radiation, which is required to initiate cancer cell death, provokes endothelial cell dysfunction, characterized by increased permeability, endothelial cell detachment from the basement membrane, and apoptosis, which often promotes inflammation and fibrosis during and after radiotherapy (Paris et al., 2001). Radiation may cause microthrombosis and increased adhesion of inflammatory immune cells to endothelial cells in the vasculature which is followed by extravasation of blood cells into the perivascular space (Wang et al., 2007). Following this, the intimal layer of the irradiated blood vessel was reported to thicken, leading to irreversible change in vasculature wall (Gujral et al., 2014; Mohammadkarim et al., 2022). Accordingly, controversial findings were reported about the impact of radiation on the angiogenesis and metastasis. Different studies indicated that damaged blood vessels may promote angiogenesis, migration of cancer cells, metastasis, and exacerbate the prognosis outcome (Girinsky, 2000; Quail and Joyce, 2013; Chaurasia et al., 2016; Hu et al., 2016; Manier et al., 2016; Viallard and Larrivee, 2017; Yadav et al., 2018; Yuan et al., 2018; Schulz et al., 2019). Despite this negative side effects in endothelium, efficient cancer elimination and better overall survival were observed during radiation treatment in many different cancers (Chaurasia et al., 2016; Hu et al., 2016; Yuan et al., 2018), suggesting that the balance towards complete cancer elimination is under control of specific component(s) in TME.

Tumor metastasis and recurrence are the main causes of death in patients with clinically advanced cancers. Metastasis and recurrence are ultimately associated with the induction of angiogenesis, which can be affected by radiation therapy (Viallard and Larrivee, 2017). Growing tumor cells require a constant supply of nutrients and oxygen, delivered through the activated growth of vasculature. A unique tumor-associated vasculature is activated through a variety of mechanisms, including an increased level of reactive oxygen spices (ROS) and activation of mechanistic target of rapamycin (mTOR) pathway (Quail and Joyce, 2013; Chaurasia et al., 2016; Hu et al., 2016; Manier et al., 2016; Schulz et al., 2019). Therefore, radiation therapy was found to stimulate not only the autophagy/apoptosis in cancer cells, but also proliferation of blood vessels near the treated tumor, increasing the degree of blood supply to tumor cells and promoting tumor metastasis (Girinsky, 2000; Yadav et al., 2018; Yuan et al., 2018).

Preliminary studies have confirmed that radiation increases the expression of vascular endothelial growth factor (VEGF) and activates pro-angiogenic signal transduction pathways (Song and Levitt, 1970; Sonveaux et al., 2003). Experimental data confirmed the association between angiogenesis and levels of VEGF. For instance, the xenografted squamous cell carcinoma cell line A431, which survived multiple rounds of irradiation (Pueyo et al., 2010), showed faster growth compared to controls *in vivo* (Pueyo et al., 2010). Significantly increased levels of VEGF, epidermal growth factor receptor (EGFR), and transforming growth factor-α (TGF-α) were detected in this study and linked to enhanced angiogenesis (Pueyo et al., 2010).

Radiation treatment may also promote metastasis in patients with squamous cell head and neck cancer (SCHNC). Conventional fractionated radiotherapy is routinely administered to stop the growth of SCHNC. The reported results showed that the microvessel density (MVD) in active SCHNC tissues after radiotherapy was significantly higher than that before radiotherapy (Zhang et al., 2003; Svagzdys et al., 2009; Harmankaya et al., 2022). The data indicated abundant angiogenesis during radiotherapy, an unexpected adverse effect. A similar finding was reported in patients with stage II/III colorectal cancers (CRCs), who received preoperative chemoradiotherapy (Zhang et al., 2017). In these patients assessed before radiotherapy, high vascular density (VD) values were found to be associated with poorer local relapse-free survival (LRFS) (Abramyuk et al., 2015; Zhang et al., 2017; Chen et al., 2021a). Following radiotherapy treatment, the proliferative index Ki-67 of the patients increased, and the VD value also exceeded the level before treatment, leading to a lower LRFS. This finding was closely related to the failure of radiotherapy in CRC patients (Koukourakis et al., 2019).

Another study assessed gene expression profiling in the paracancerous tissues of rectal cancer patients who received radiotherapy. The study reported that the expression of angiogenesis-promoting genes and MVD were significantly increased, indicating activation of angiogenesis by radiation (Gil Marques et al., 2019). In lung cancer tissues, radiotherapy triggered the hypoxia-inducible factor 1α (HIF-1α) and VEGF pathway. Alternatively, inhibition of this pathway reduced radiotherapy-induced tumor angiogenesis, indicating the importance of the HIF-1α/VEGF pathway for radiation-related stimulation of vascular growth (Zhu and Zhang, 2018). Conclusively, the tumor cells that were not eliminated by radiation, may progress towards a more advanced malignancy which is supported by abundant blood vessel formation. Therefore, the undesirable side-effects of radiotherapy were ultimately linked to tumor recurrence and metastasis.

**FIGURE 2:**
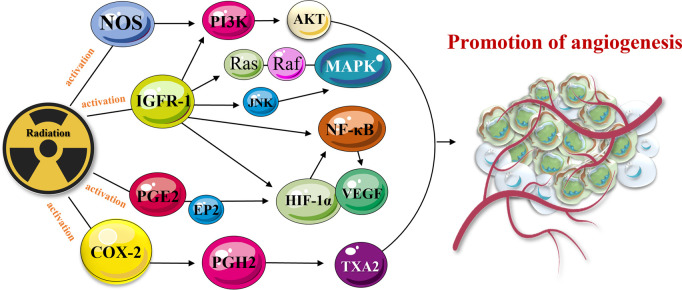
TME-activated COX-2 and PGE2 signaling pathways mediate cancer progression. The pathway is linked to angiogenesis via a complex molecular network. Radiation-activated COX-2 and secreted PGH2 cause the production of TXA2 and promote angiogenesis. Released PGE2 activates the expression of HIF-1α and VEGF which also promote angiogenesis and cancer resistance/metastasis.

Irradiation was also found to activate various cell membrane tyrosine kinase receptors, such as insulin-like growth factor-1 receptor 1 (IGFR-1) (Husain et al., 2010; Owens et al., 2013; Kilpatrick and Hill, 2021). The receptor is actively involved into autocrine signaling processes and was shown to promote cell-to-cell communication, cell growth, and division (Husain et al., 2010; Owens et al., 2013; Kilpatrick and Hill, 2021). Activated IGFR-1 transmits signals through multiple downstream effectors, including transcription factor NF-κB and associated signaling networks, such as rat sarcoma (Ras) pathway, phosphoinositide 3-kinase (PI3K), mitogen-activated protein kinase (MAPK), c-Jun N-terminal kinase (JNK), Fas cell surface death receptor (Fas-R), and tumor necrosis factor-α (TNF-α) signaling network (Sorolla et al., 2020). Many of radiation-induced mediators directly or indirectly promote the expression of VEGF/IGFR-1 and angiogenesis (Figure 2). For instance, radiation was found to activate cyclooxygenase-2 (COX-2), nitric oxide synthase (NOS), EGFR, and IGFR-1 pathways (Eldesoky et al., 2011; Banys-Paluchowski et al., 2018). The pathways are represented by complex networks and can stimulate angiogenesis via intermediate signaling molecules. Radiation-activated COX-2 can induce the production of prostaglandin H2 (PGH2). Following this, PGH2 triggers thromboxane A2 (TXA2) and promotes vascular growth (Mitryayeva et al., 2019; Anoopkumar-Dukie et al., 2020). Radiotherapy was also shown to activate production of prostaglandin E2 (PGE2) which promotes the expression of HIF-1α and VEGF (Kovacs et al., 2019). PGE2 can also bind to prostaglandin E2 receptor subtype 2 (EP2) and reinforce VEGF signaling (Figure 2) (Li et al., 2019).

Radiation was also found to activate NOS (Holotiuk et al., 2019; Wei et al., 2019), which is responsible for the generation of nitric oxide (NO), induction of HIF-1α expression (Masoud and Li, 2015), activation of PI3K/Akt/FRAP or PI3K/Akt/mTOR pathways, and angiogenesis (Sharifpanah et al., 2019). Similar effects (activation of angiogenesis and cancer recurrence/metastasis) were achieved when radiotherapy activated the EGFR network (Alique et al., 2020). EGFRs are coupled to the induction of VEGF expression via Ras/MEK/MAPK, Ras/MEK/ERK/MNK, PI3K/Akt/FRAP, PI3K/Akt/mTOR and other kinase signaling pathways (Zhu et al., 2020). The signaling pathways, including PI3K/Akt/FRAP, PI3K/Akt/mTOR, and MEK/ERK/MNK, can be also activated by radiation and IGFR-1. HIF-1α and VEGF are also triggered by above-mentioned networks, leading to blood vessel growth (Masoud and Li, 2015) (Figure 2). Furthermore, in the hypoxic microenvironment, which is commonly detected within solid tumor tissues (Alique et al., 2020), low-dose irradiation activates VEGF receptor 2 (VEGFR2) (Marques et al., 2020). The cell damage, caused by radiation, increases the degree of hypoxia, and stimulates the secretion of VEGF-A by tumor cells; thereby, stimulating angiogenesis via VEGF pathway (Jabbari et al., 2019).

Aside from VEGF, TGF-β also connects multiple signaling pathways, participates in the promotion of tumor angiogenesis, stimulates CAF activation, activates HIF-1 signaling, and is also involved in the activation of T cells and dendritic cells (DCs) in TME (Barcellos-Hoff, 2022). Radiation was found to upregulate TGF-β expression in tumor cells. TGF-β indirectly affects angiogenesis through induced expression of HIF-1α/VEGF (Rodriguez-Ruiz et al., 2020). In conclusion, radiation may stimulate and promote angiogenesis, employing HIF-1α/VEGF and other signaling pathways.

### 2.2 Combined application of radiotherapy and targeted anti-tumor vascular therapy

Progressive growth of solid tumors leads to the generation of hypoxic TME areas due to insufficient vascular distribution. The hypoxic TME was associated with tumor radioresistance (Chen et al., 2016). To increase the radiosensitivity of tumor cells, several effective anti-angiogenic agents (vascular inhibitors) were generated. These substances can specifically target and inhibit the growth of new blood vessels, regulate the blood flow and oxygenation of cancer tissues (Chen and Xu, 2015; Siemann et al., 2017; Uribesalgo et al., 2019). Vascular inhibitor therapy can block tumor growth to a certain extent. The therapy was limited by the development of drug resistance, increased levels of hypoxia, and various obstructions to effective drug delivery. Unfortunately, the overall survival after treatment with vascular inhibitors in clinical practice is relatively poor (Lin et al., 2016). However, combined treatment with angiogenesis inhibitors and radiotherapy were found to enhance the radiosensitivity of endothelial cells (Blomberg et al., 2020), attenuate radiotherapy-induced angiogenesis, and prevent tumor recurrence. Experimental and clinical studies have shown that radiotherapy combined with vascular inhibitors has significantly better anti-cancer effect (Kanthou and Tozer, 2019).

Several molecular targets have been selected to improve effects of radiotherapy, including purine/pyrimidine endonuclease 1 (apurinic/apyrimidinic endonuclease 1, APE1), the enzyme with DNA repair functions (Teicher et al., 1995; Kidoikhammouan et al., 2012; Gu et al., 2013; Xi et al., 2013). It has been found that X-rays can induce both the expression of APE1 and VEGF in A549 cells (Gu et al., 2013; Xi et al., 2013). Inhibition of APE1 expression significantly attenuated radiation-induced endothelial cell migration and greatly reduced the formation of capillary-like structures (Gu et al., 2013). The sensitivity of the tumor to radiotherapy was significantly enhanced by co-treatment with another anti-angiogenic agent TNP-470 (minocycline; methionine aminopeptidase-2 inhibitor) (Kidoikhammouan et al., 2012). TNP-470 demonstrated efficient anti-angiogenetic properties in rat subcutaneous gliosarcoma model *in vivo* (Teicher et al., 1995).

Bevacizumab, a VEGF inhibitor (a recombinant humanized monoclonal antibody against VEGF), has been used in clinical trials for the treatment of head and neck cancer (HNC), where it has significantly improved radiation therapy (Ahn et al., 2018). Combined regimens with bevacizumab and erlotinib (EGFR blocker) were used during chemoradiotherapy (CRT) protocols to treat patients with advanced HNC. The survival rate of these HNC patients with complete remission (cure rate, CR) reached 96%, while 3-year locoregional control rate was 85%, distant metastasis-free survival rate was 93%, and overall survival rate was 86% (Yoo et al., 2012). Postoperative chemotherapy and pelvic IMRT plus bevacizumab in patients with high-risk endometrial cancer were well tolerated and improved 2-year overall survival (Viswanathan et al., 2015). A similar combined regimen was also tested in patients with extremity soft tissue sarcomas (ESTS). The patients were treated with pazopanib (another monoclonal antibody against VEGF), combined with radiotherapy. The results showed that pathological complete remission was achieved in 4 out of 10 patients (Haas et al., 2015). Drug toxicity and side effects were acceptable when conventional fractionated radiotherapy was combined with sorafenib (VEGF pathway inhibitor; small molecule tyrosine kinase inhibitor) (Zhao et al., 2010; Chen et al., 2014).

Endostatin, an endogenous inhibitor of angiogenesis, is a 20-kD proteolytic fragment of type XVIII collagen (Digtyar et al., 2007). Endostatin can bind the integrin α5β1 in the endothelial cell membranes, leading to the activation of Src-kinase signaling. Combined with concurrent chemoradiotherapy (CCRT), endostatin was used for the treatment of patients with unresectable stage III Non-Small Cell Lung Cancer (NSCLC). The results showed considerable improvements in survival and local cancer control rates (Bao et al., 2015). A recent study with PEGylated recombinant human endostatin indicated the potential of this anti-angiogenic substance for the treatment of pancreatic and gastric cancers (Guo L. et al., 2023). Endostatin can not only block cancer-induced VEGF expression, but also demonstrated a better safety profile (compared to bevacizumab) in small clinical studies (Mendez-Valdes et al., 2023), although further testing with combined radiotherapy is required.

Erlotinib (also known as OSI-774 or Tarceva), is an anti-EGFR antibody that was approved as an anticancer drug by the US-FDA and tested as a treatment for NSCLC and pancreatic cancer (Feng et al., 2014). For patients with esophageal squamous cell carcinoma who cannot tolerate chemoradiotherapy, the administration of erlotinib combined with radiotherapy was more tolerable and effective (Zhai et al., 2013). The targeted search for improved inhibition of angiogenesis continues and resulted in the design of more efficient inhibitors of EGFR signaling (Shah et al., 2023), although clinical testing is required before those drugs can be implemented. Conclusively, the previous studies showed that local control rate and overall survival rate of patients who were treated with angiogenesis inhibitor combined with radiotherapy are significantly improved compared with radiotherapy alone. The application of vascular inhibitors also reduced radiation-induced damage to normal tissues and improved patients’ treatment tolerance. Moreover, the combined treatment protocol significantly reduced tumor recurrence, although further testing is warranted. It is foreseeable that combined radiotherapy and adjuvant therapy with anti-angiogenic substances will play an increasingly important role in tumor therapy.

## 3 Effects of radiation therapy on cancer-associated fibroblasts (CAF)

### 3.1 Radiotherapy affects CAF-regulated network

CAFs are represented by highly heterogeneous cell populations which account for about 50% of the total number of all TME cells (Butti et al., 2023; Wieder, 2023). Therefore, CAFs are often defined as stromal TME cells (De et al., 2021; Kochetkova and Samuel, 2021; Mor et al., 2022). It has been suggested that quiescent fibroblasts can be transformed into CAFs in tumor tissue. Undergoing epithelial/endothelial-mesenchymal transition (EMT), bone marrow-derived differentiated mesenchymal stem cells, and epithelial or endothelial cells were also linked to the generation of CAFs (Kamali Zonouzi et al., 2021; Kochetkova and Samuel, 2021; Loh and Ma, 2021; Simon and Salhia, 2021). CAFs were shown to play an important regulatory role in the TME, where these cells promoted cancer occurrence, metastasis, and recurrence (Barbazán and Matic Vignjevic, 2019; Kashima et al., 2019). CAFs can express and secrete a variety of cytokines that promote tumor cell proliferation, invasion, and metastasis (Peng et al., 2021; Chen et al., 2022; Li et al., 2023; Morgan et al., 2023). For instance, isolated from tumor-stromal junctions CAFs were able to activate EMT in tumor cells, leading to increases in cancer cell migration (Asif et al., 2021; Majidpoor and Mortezaee, 2021; Stuelten and Zhang, 2021; Taeb et al., 2022; Pei et al., 2023). Numerous pro-angiogenic factors and matrix metalloproteinases (MMPs), which activate angiogenesis and lymphangiogenesis, can be secreted by CAFs (Wei et al., 2017; Cadamuro et al., 2019; Chen et al., 2021b; Ambrosetti et al., 2022; Chiavarina et al., 2022; Watanabe et al., 2022). Notably, CAFs can also secrete anti-inflammatory chemokines and modulate immune anti-cancer responses, thus, promoting carcinogenesis (Zheng et al., 2021a; Zheng et al., 2021b; Kobayashi et al., 2021). The presence of CAFs in TME was linked to the poor prognosis and development of drug resistance (Chen et al., 2021b; Dong et al., 2021; Kamali Zonouzi et al., 2021; Ambrosetti et al., 2022).

A very limited number of studies addressed the impact of radiotherapy on the level of CAFs in TME. It has been demonstrated that CAFs, which were isolated and cultured from lung cancer tissue, can survive radiation exposure, but their invasive ability may be lost (Hellevik et al., 2012). The CAF-based analysis suggested that radiation activates the overexpression of integrins α2 and β1, thereby attenuating the migratory ability of CAFs (Park et al., 2008; Nam et al., 2013). Subsequent clinical studies confirmed the important role of β1 integrin expression in tumor cells surviving after radiotherapy (Park et al., 2008). Further studies have shown that tumor stroma mediates the development of radioresistance in cancers, and β1 integrin is a key link involved in this process (Carracedo et al., 2010; Eke et al., 2012; Ahmed et al., 2013; Broustas and Lieberman, 2014; Wu et al., 2015; Zhang et al., 2018; Yokosaki and Nishimichi, 2021; Park et al., 2023).

**FIGURE 3:**
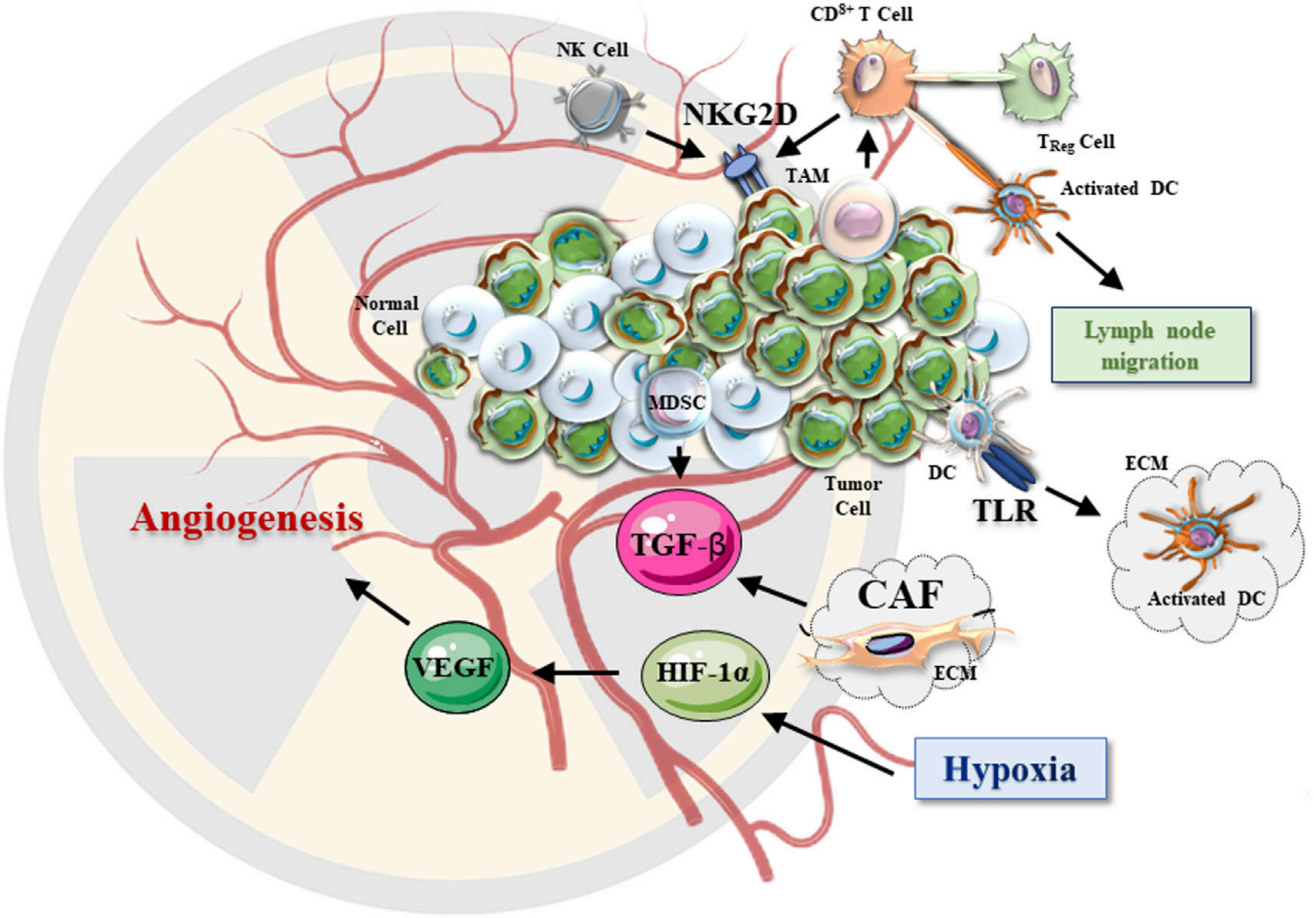
The effects of radiotherapy on TME. After irradiation, TME undergoes a series of changes. The hypoxic environment of irradiated solid tumors triggers the HIF-1 signaling pathway. The secreted VEGF and TGF-β promote tumor growth and angiogenesis. CAFs in the tumor extracellular matrix are activated upon irradiation and lead to changes in the secretion of various growth factors. After irradiation, NKG2D signaling is also enhanced, resulting in improved (anti-cancer) cytotoxic effects of NK and CD8^+^ T cells. Increased proinflammatory factors, DAMP-related TLRs may activate DCs, which further activate the anti-cancer effect of T cells in TME.

Radiation was suggested to enhance the secretion of growth-promoting growth factors and ECM regulators (TGF-β and MMPs) from CAFs (Figure 3).

TGF-β can be secreted by CAFs and directly stimulate tumor immune evasion and activate the HIF-1 signaling pathway. MMPs were also shown to promote angiogenesis and tumor cell invasion and metastasis (Dancea et al., 2009; Hneino et al., 2012; Neuzillet et al., 2015). Considering that radiotherapy was shown to promote EMT, tumor invasion, and migration, it is logical to suggest that CAFs may be induced by radiation. For instance, radiotherapy promoted the expression of MMP-2 (via activated EGFR/Akt pathway) and enhanced the invasive ability of glioma cells (Park et al., 2006). Increased MMP-9 expression by was also associated with higher invasive ability of hepatocellular carcinoma cells (Cheng et al., 2006). However, it remains to confirm whether CAFs are involved in this process and activated by radiotherapy.

### 3.2 Combined effects of radiotherapy and CAF-targeting therapy

According to the mutation analysis, CAFs are genetically more stable than tumor cells, making them a better target for therapy. CAF genes, which are responsible for the secretion of TGF-β, MMPs, hepatocyte growth factor (HGF), and tenascin-C (Tn-C), were identified as promising therapeutic targets (Guo S. et al., 2023; Greimelmaier et al., 2023; Li and Mu, 2023; Yang et al., 2023). The combined treatment protocol with radiotherapy and TGF-β inhibitors chemotherapy was found to be effective for attenuation of tumor cell proliferation and spreading (Bouquet et al., 2011). It was also found that MET (mesenchymal epithelial transition factor) inhibitors enhanced the efficacy of radiotherapy and attenuated the invasive activity of tumor cells. This additive effect was mediated by HGF secretion (De Bacco et al., 2011). However, there have been no clinical trials that tested the use of HGF and MET inhibitors combined with radiotherapy. Therefore, it is warranted to verify this novel approach in cancer therapy.

Other CAF-linked molecular targets were also identified, although not completely tested in clinics. For instance, during the activation of CAF, the high expression of Tn-C in the cell matrix may allow the iodine-131-labeled anti-Tn-C antibody to enter the matrix precisely into cancer cells. It was suggested that this cell-targeted approach should be tested in clinical settings. (Reardon et al., 2002). Treatment effects of monotherapy clinical trials were found to be suboptimal, possibly due to the broad-spectrum activity of MMP inhibitors. Relevant preclinical studies have found that MMP inhibitors with higher specificity can improve the efficacy of radiotherapy (Waas et al., 2005; Heath et al., 2006; Olivares-Urbano et al., 2020; Waller and Pruschy, 2021), but further research is needed.

## 4 Radiation therapy and cancer-associated exosomes

### 4.1 Effects of radiotherapy on the secretion and composition of exosomes

Tumor cells maintain the development and stability of their TME by secreting various growth-promoting factors and extracellular matrix filaments. Vesicle-encapsulated structures (exosomes) with a diameter of up to 50–150 nm are often released by cancer cells (Gyorgy et al., 2011). The exosomes were found to contain various microRNAs, proteins, and lipids (Malla et al., 2017). The released structures are generated by the initiation membrane, undergo endocytosis to form polycystic bodies, and are secreted to the extracellular liquids after maturation. The released exosome may be absorbed by recipient cells (phagocytosis or membrane fusion), leading to the activation of various physiological processes in the targeted cells and tissues. Accordingly, cancer-released exosomes were shown to direct intercellular signal transmission (Bang and Thum, 2012), and immune response (Robbins and Morelli, 2014), and facilitate the cancer escape from apoptosis (Miksa et al., 2009).

The two significant factors, which affected the release of exosomes, were linked to radiation-induced DNA damage and activation of p53-related pathways (Tarasov et al., 2021; Tan et al., 2023). Radiotherapy is designed to cause DNA damage in cancer tissues. Moreover, it also activates many related pathways, including the p53 network. The tumor suppressor protein p53 regulates the transcription of many genes, including the genes responsible for the communication between adjacent cells. The activation of p53 pathway was found associated with the regulation of endocytic trafficking and the exosome secretion during apoptosis (Yu et al., 2006; Lakoduk et al., 2021). Increased cancer cell invasion and resistance were observed in cells with mutant p53 which influenced expression and function of genes responsible for vesicle trafficking and recycling of growth factor receptors. Formation and secretion of exosomes are also regulated by p53 in the TME (Yu et al., 2006; Lakoduk et al., 2021).

The composition of exosomes is defined by the cell type and is strongly affected by radiation. Several experimental studies confirmed that tumor cells treated with ionizing radiation generate exosomes with various and complex content. For instance, human brain astrocytoma cell U-87MG, irradiated with 4 Gy for 48 h, released exosomes with connective tissue growth factor and IGF-binding proteins (Arscott et al., 2013). Prostate cancer cells 22Rv1, exposed to 4 Gy radiation (for 96 h), released a higher amount of B7-H3 proteins (Lehmann et al., 2008). In samples from prostate cancer patients, the content of heat shock protein 72 was found to increase after radiotherapy (Hurwitz et al., 2010). After irradiation of human head and neck squamous cell carcinoma cells using the Feature Aggregate Depth Utility (FaDu) (2 Gy, 18 h) approach, it was registered that levels of proteins involved in the regulation of translation and transcription (cell cycle regulators) were increased (Chung et al., 2021). Alternatively, the expression levels of apolipoproteins and immunoglobulins decreased (Jelonek et al., 2015). These findings indicated that the effects of ionizing radiation can be reflected in changes in the exosome content, which may be used in the post-treatment diagnostics.

### 4.2 Exosome functions and radiotherapy

Exosomes are functional elements of normal physiology and can be released by normal cells. Moreover, exosomes mediate the activation of immune responses. For instance, the major histocompatibility complex (MHC)-peptide complexes carried by exosomes can bind to cognate T cell receptors and activate CD4+/CD8+ T lymphocytes (Nolte-'t Hoen et al., 2009; Xie et al., 2010; Admyre et al., 2006). This is the common mechanism of antigen presentation which initiates the body’s immune response. Exosomes may also interfere with the body’s immune system and promote tumor growth. Several recent studies have shown that cancer-released exosomes can slow down the proliferation of NK cells (Liu et al., 2006) or CD^4+^/CD^8+^ T cells (Clayton et al., 2007), promote the mutation of myeloid cells (Valenti et al., 2006) and regulatory T cells (Szajnik et al., 2010).

The ability of exosomes to activate immune responses relies on the expression of membrane antigens they carry and the physiological state of the target cells (Segura et al., 2005; Bhatnagar et al., 2007). Exosomes secreted by tumor cells may also carry immunosuppressive peptide complexes. Accordingly, tumor-released exosomes have also been implicated in tumor spreading. Irradiation-induced exosome-specific changes were found to trigger nerve growth factor (NGF)-tyrosine protein kinase (Trk) A and focal adhesion kinase (FAK) signaling pathways in brain cancer patients (Wang H. et al., 2021). Activation of Trk ultimately promotes brain glial plasma cell migration and metastasis (Arscott et al., 2013). Trk is also involved in the regulation of intracellular trafficking (Scott-Solomon et al., 2018), required for the establishment of cell-to-cell communications in TME (Okimoto and Bivona, 2016).

Exosomes regulate the information exchange between tumor cells and TME by transmitting unique signals related to apoptosis, division, and growth to target cells (Ludw et al., 2012). Ionizing radiation can significantly affect the exchange of information between cells through the conformation of different signal transduction systems. The release of specific exosomes with pro-survival cargo affects the biological behavior and therapeutic effects in tumors and TME. Radiation-related changes in cell-to-cell communications have been shown to regulate radiotherapy responses in the directly irradiated cells and surrounding unirradiated cells via the employment of exosomes and their cargo (Moore et al., 2005; Facoetti et al., 2006; Burr et al., 2010). Experimental data demonstrated the increased gene instability which is translated into changed content of exosomes (modified RNAs, microRNAs, and protein cargo) in irradiated breast cancer (Al-Mayah et al., 2012; Al-Mayah et al., 2015) and other cells (Malla et al., 2017). Furthermore, radiation may promote the release of exosomes with specific cargo (microRNAs) which facilitate the development of radioresistance and radiation-induced bystander effects (RIBEs) (Yang et al., 2022). Additionally, the uptake of exosomes by irradiated cells was found to be also affected by radiation. It has been shown (Hazawa et al., 2014) that human bone marrow-derived cells exposed to ionizing radiation increased exosome uptake through CD29/CD81 complex formation and p38 mitogen-activated protein kinase-dependent endocytosis and pinocytosis. In conclusion, considering the role of exosomes and their cargo in the regulation of cancer cell growth and survival, it has been suggested to employ exosomes as a target for anti-cancer treatment (Ni et al., 2019).

## 5 Radiotherapy effects on cellular components of TME

### 5.1 Radiotherapy enhances the anti-cancer immune responses in TME

Tumor immunity is designed to deliver anti-tumor effects via mobilization of the host’s natural defense mechanism and secretion of endogenous anti-cancer substances (Wattenberg et al., 2014). Accordingly, tumor immunotherapy, which was introduced in the 1980s, has become the fourth tumor treatment method following surgery, radiotherapy, and chemotherapy. In 2013, it was indicated as the leading technological breakthrough achievement in cancer therapy (Couzin-Frankel, 2013). Currently, a large number of drugs have been developed and tested in clinics (Table 1), although only some of them have been tested in combination with radiotherapy. Notably, radiotherapy can promote the body’s natural anti-tumor immunity. Irradiation causes DNA damage and apoptosis in malignant cells (Golden et al., 2012). The triggering of apoptosis and necrosis can induce the innate immune response in the TME, causing the release of cytokines and chemokines. The released products can recruit macrophages, NK T cells, regulatory T cells, and myeloid suppressor cells (MDSC) (Er et al., 2007; Gallo and Gallucci, 2013). The reported anti-tumor effect in the remote non-radiation area induced by radiation (abscopal radiation effect) indicated that radiotherapy promoted immune anti-cancer responses (Lhuillier et al., 2021). Accordingly, radiation treatment of immunodeficient tumor-bearing nude mice did not activate the abscopal radiation effect, indicating the need for robust immune effectors to trigger the response (Demaria et al., 2004; Lumniczky and Safrany, 2015).

**TABLE 1:** Overview of drugs targeting TME for radiotherapy.

Targeting mechanism	Drug	Target protein/gene
Angiogenesis/Hypoxia	Nimorazole	HIF-1
	Albumin-MnO2	HIF-1
	Acriflavine	HIF-1
	Aflibercept	VEGF, PIGF
	AMG386	ANG1, ANG2
	Endostatin	VEGF, TGF-β, HIF-1
	AMD3100	CXCL12, CXCR4
	Cilengitide	Integrins
	Vitaxin	Integrins
	Volociximab	Integrins
	Bevacizumab	VEGF
	Erlotinib	VEGF
	Pazopanib	VEGFR, PDGFR
	Apatinib	VEGFR
	Axitinib	VEGFR
	Linifanib	VEGFR, PDGFR
	Vandetanib	VEGFR, EGFR
	Cabozantinib	VEGFR
	Regorafenib	VEGFR, PDGFR
	Sunitinib	VEGFR, PDGFR
	Lenvatinib	VEGFR, PDGFR
	Sorafenib	VEGFR, PDGFR
	Brivanib	VEGFR, FGFR
	Nintedanib	VEGFR, PDGFR
	Motesanib	VEGFR, PDGFR
	Cediranib	VEGFR
CAF/Fibrosis	Nintedanib	PDGF, VEGF,
	Imatinib	TGF-β, PDGF
	Nilotinib	TGF-β, PDGF
	Dasatinib	TGF-β, PDGF
	Vismodegib	Smoothened receptor
	Saredigb	Smoothened receptor
	Sonedegib	Smoothened receptor
	Suramin	PDGF, EGF, TGF-β, FGF
	SD-208	TGF-βRI
	Simtuzumab	LOXL2
	81C6	TN-C
	F16SIP	TN-C
Immune Response	Ipilimumab	PD-1, CTLA4
	Imiquimod	PD-1,TLR7
	Nivolumab	PD-1
	Durvalumab	PD-1
	Pembrolizumab	PD-1
	Pidilizumab	PD-1
	AMP-224	PD-1
	REGN2810	PD-1
	Atezolizumab	PD-L1
	Avelumab	PD-L1
	Durvalumab	PD-L1

There is increasing evidence that radiotherapy can re-activate cancer surveillance and enhance innate and adaptive immune responses against tumors. A common hypothetical theory is that local radiotherapy induces immunogenic cell death, resulting in the activation of pro-inflammatory TME. The pro-inflammatory condition is characterized by the release of tumor antigens and damage-associated pattern molecules (DAMPs) from the dead cells (Ahmed et al., 2014; Weichselbaum et al., 2017). Radiation therapy can also induce the expression of various chemokines, leading to the enrichment of T cells in the TME. These factors promote the activation of antigen-presenting cells (Oh et al., 2020). Several *in vitro* and *in vivo* experiments indicated that radiation-induced the production of pro-inflammatory cytokines IL-1β and tumor necrosis factor (TNF-α). The secretion of these cytokines promoted dendritic cell (DC) maturation (Shi et al., 2003). DCs are bone marrow-derived cells that are greatly affected by radiation-induced alterations in the TME (Kim et al., 2004). Radiation has been shown to increase the amount of tumor-associated DCs, enhance the homing of these cells to lymph nodes, and induce the maturation of DCs and their ability to present antigens (Cummings et al., 2009).

Preclinical studies have demonstrated that radiation-induced tumor antigen release promote antigen-presenting cells (APCs) to migrate to lymph nodes, where T cells are primed to initiate systemic responses (Aggarwal, 2023). The National Cancer Institute (NCI) report shows that radiation can alter the phenotype of tumor cells, leading to upregulation of cell surface molecules, thereby broadening the antigens available for presentation and making tumors more susceptible to T cell-mediated anti-tumor effect (anti-cancer surveillance) (Roses et al., 2014). Aside from T lymphocytes, NK cells are also an important part of tumor surveillance. Involvement of the NK Group 2 member D (NKG2D) receptor was reported (Figure 3). Radiation increases the expression of NKG2D ligands in several human tumor cell lines, suggesting that NK cell-based therapies should be developed and explored in clinical trials (Sayitoglu et al., 2020; Wang J. et al., 2021; Khan et al., 2021).

However, some factors may impede the radiotherapy effects and promote resistance. For instance, changes in the level of TGF-β in patients with esophageal cancer after radiotherapy were associated with the development of resistance and were suggested to be explored as a predictor of pulmonary fibrosis after radiotherapy. Accordingly, the inhibition of TGF-β is crucial to improve the efficacy of radiotherapy (Biswas et al., 2007; Biswas et al., 2017). The dual role of radiotherapy and the development of resistance will be described below.

### 5.2 Undesirable effects of radiotherapy: immunosuppression

A variety of inhibitory immune cells has been found in TME and are associated with the development of resistance and cancer progression. The inhibitory cells are often represented by the regulatory T cells (Treg), macrophages, and MDSCs (Fridman et al., 2012). The Treg cells group includes T cells with significant immunosuppressive effects, characterized by the expression of Foxp3, CD25, and CD4 (Hariyanto et al., 2022). Those Tregs can suppress the immune response of other immune cells and are the main mediators of self-tolerance (Sakaguchi et al., 2020). Under normal conditions, the low levels, or dysfunctions in Tregs result in the development of autoimmune diseases. The majority of immune cells, such as T cells, B cells, and NK cells, can recognize and protect the body from the “non-self” agents, generate an immune response, and remove harmful substances. Treg cells are destined to regulate immune responses, stop the propagation of exaggerated immune signals, regulate immune homeostasis, and prevent autoimmune diseases (Collison et al., 2007; Haddadi and Negahdari, 2022). Specifically, Treg cells can secrete anti-inflammatory cytokines TGF-β and IL-10 in the TME. Those cytokines inhibit the activities of effector T cells and activate other suppressor cells, including MDSCs (Facciabene et al., 2012; Bu et al., 2013). Therefore, Treg cells may serve as immune system suppressors.

It has been observed that the number of Treg cells in tumor tissues and immune organs is often significantly increased in patients receiving clinical radiation therapy (Kachikwu et al., 2011). Experiments showed that the expression of the cytotoxic T-lymphocyte-associated antigen 4 (CTLA-4) in Treg cells increased within 72 h after whole-body irradiation. Simultaneously, the production of TGF-β (Moreau et al., 2022) and IL-10 by Treg cells was also increased (Balogh et al., 2013). This data suggested that Treg cells may enhance immunosuppressive TME after irradiation (Persa et al., 2015). Therefore, to improve anti-cancer therapy, it was suggested to target Treg cells and the associated immunosuppressive molecules (TGF-β and CTLA-4) (Whiteside, 2012; Sanjabi et al., 2017). Aside from Tregs, MDSCs and tumor-associated macrophages (TAMs) play an important role in promoting tumor angiogenesis which provides substantial pro-survival support to the irradiated cancer tissues (Figure 3) (Sica et al., 2008). MDSCs may also promote the development of radiotherapy resistance (Persa et al., 2015). After irradiation, the accumulation of MDSCs in the tumor stroma was found to be elevated (Lehmann et al., 2008). Furthermore, radiation altered the expression levels of TAM-produced chemokines, thereby altering the regulation of T-cell infiltration in cancer tissues (Inoue et al., 2004; Beach et al., 2022). However, it is warranted to clarify how the TME is involved in the regulation of radiation-induced TAM recruitment and which MDSC and TAM cell populations should be targeted to improve the anti-cancer treatment.

### 5.3 Radiotherapy and immune checkpoint inhibitors: a combined approach

**FIGURE 4:**
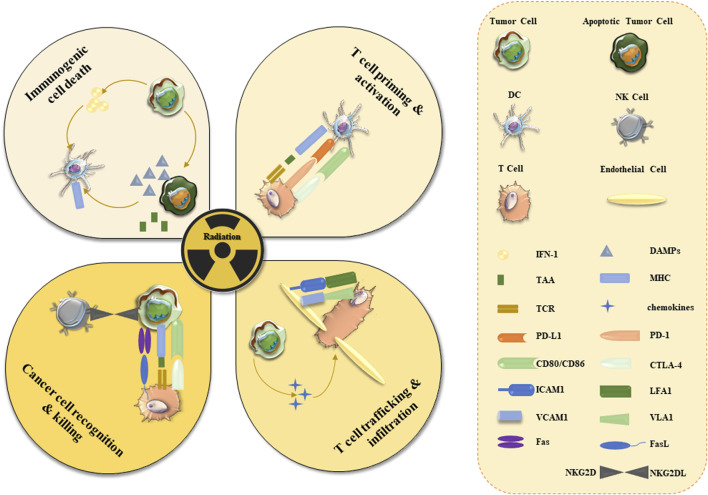
Activation of anti-cancer immune responses in TME by radiotherapy. Radiotherapy induces cancer cell apoptosis and necrosis which are followed by the release of cancer cell antigens. Apoptosis leads to the increased expression of the damage-related molecular pattern (DAMPs) proteins and interferon-1 (INF-1), which can activate various immune cells (DC, NK, and T cells). Tumor-derived antigens trigger NK/T cell recognition and elimination of tumor cells. Expression of inhibitory effectors (PD-1 and CTLA-4) can prevent tumor elimination and should be targeted by ICIs. Angiogenesis (marked by the expression of VCAM) may slow down cancer eradication. Radiotherapy stimulates immune surveillance and triggers the expression of MHC, Fas ligands/receptors, and NKG2D ligands. Abbreviations: CD - cluster of differentiation; ICAM1—intracellular adhesion molecule 1; L-ligand; LFA1 - leukocyte function-associated antigen-1; TAA-tumor-associated antigens; TCR - T cell receptor; VCAM1 - vascular adhesion molecule 1; VLA1-vascular integrin alpha 1.

The immune system has multiple checkpoints that can be engaged to stimulate or inhibit certain T-cell functions. Immune checkpoint inhibitors (ICIs) were shown to play an important role in overcoming tumor immune tolerance, providing a new treatment method in cancer therapy (Bagchi et al., 2021). The application and development of ICIs represent an attractive method in the current cancer immunotherapy (Galluzzi et al., 2020). During the last decade, ICI-based drug research has made fruitful progress (Gubin et al., 2014; Powles et al., 2014). The immune system is a key component of the abscopal radiation effect of radiotherapy (Postow et al., 2012). Local radiotherapy is tightly linked to the activation of various immune system responses, including T cell priming and activation, immunogenic cell death, T cell trafficking, and tissue infiltration, and cancer recognition and elimination (Figure 4).

Radiation induces immunogenic cell death of tumor cells, which activates the immune system by presenting tumor antigens to cytotoxic T lymphocytes (Krysko et al., 2012). For instance, radiotherapy can promote the migration of effector CD8^+^ T cells and enhance the function of effector T cells by inducing the TME-linked production of pro-inflammatory chemokines and cytokines (Burnette et al., 2011). Antigen expression and presentation, and enhanced effector T cell function provide a plausible underlying rationale for immune-mediated radiation abscission effects (Lee et al., 2020). Therefore, the combined application of radiotherapy and ICIs may produce a better outcome, although it remains to be confirmed in clinical trials.

At present, ICI drugs were approved and are being clinically tested as a treatment for solid tumors, including melanoma and non-small cell lung cancer (Borghaei et al., 2015; Larkin et al., 2015). ICIs for CTLA-4 and programmed death (PD) ligand-1 (PD-1/PD-L1) targets are the most studied substances which indicated promising results. For instance, the CTLA-4 pathway is an immunosuppressor for CD8^+^ T cells. Combined with radiation therapy, CTLA-4 inhibitor drugs, such as ipilimumab, were found to be highly effective (Demaria et al., 2013). Experimental studies have shown that combined radiation and CTLA-4 inhibitor treatment effectively inhibited lung metastasis of primary breast cancer in mice (Demaria et al., 2005). Notably, radiotherapy combined with CTLA-4 mAb was more effective than either modality used alone (Dewan et al., 2009). It has been suggested that combination therapy is more effective in preclinical model studies because radiation can increase the expression level of the NKG2D protein in tumor cells, making the cancer cells more vulnerable to NK cell attack (Demaria et al., 2013). Recently, several studies have explored and summarized the progress and challenges of ICI drug application during immunotherapy. Novel immunosuppressive pathways were targeted by various therapeutic molecules, such as LAG-3, TIM-3, TIGIT, VISTA, and B7-H3 (Franzin et al., 2020; Marin-Acevedo et al., 2021). Application of novel substances and/or use of novel methods have both specific benefits and challenges, including increased toxicity, which should not be neglected (Marin-Acevedo et al., 2021). For instance, the serious renal side effects of ICI drug application, such as acute kidney injury (AKI) and acute tubulointerstitial nephritis (ATIN), were reported. Several strategies for prevention and management of these complications were proposed (Franzin et al., 2020) and requires future assessment.

PD-L1, whose expression is undetectable in most normal tissues, can be induced in various cell types by inflammatory cytokines (Minn, 2015). PD-L1 is expressed in various solid tumors, and its presence is an important indicator of whether a patient responds to PD-1/PD-L1 inhibitors (Ribas, 2012; Topalian et al., 2012; Postow et al., 2015; National Library of Medicine, 2018). Using tumor-bearing mice as a model, combined treatment with radiotherapy and a PD-L1 inhibitor led to significant tumor regression (Lan et al., 2021). It was further observed that the infiltration of CD8^+^ T cells resulted in a significant decrease in the number of MDSCs in the tumor tissues treated with the combined therapy method (Raskov et al., 2021; Reina-Campos et al., 2021). It has been noted that the level of CD8^+^ T cells in the host decreases when the level of MDSCs increases. PD-L1 inhibitors can restore anti-cancer T-cell function and increase sensitivity to apoptosis in tumors (Gabrilovich et al., 2012; Kumar et al., 2016). Stereotactic radiotherapy combined with a PD-1 inhibitor significantly improved survival compared with monotherapy in a mouse model of brain tumors (Zeng et al., 2013; Stessin et al., 2020). A higher level of CD8^+^ T cells and a lower degree of Treg cell infiltration were also detected in the tumors treated with combination therapy (Zeng et al., 2013). Research data indicated that radiotherapy combined with PD-1 inhibitors significantly increased the number of memory CD8^+^ T cells (Sharabi et al., 2015). In conclusion, radiotherapy can activate local and systemic immune responses required for tumor elimination. Therefore, the combination of radiotherapy and immunotherapy can enhance the body’s innate and adaptive anti-cancer immunity essential for patient survival. However, it remains to understand how to minimize the side effects of combination therapy and design the best optimal protocol for clinical applications.

## 6 Conclusion and future perspectives

Anti-tumor combined therapies are the current focus in the development of effective cancer treatment for advanced malignancies. This study provides insights into the mechanisms of radiotherapy effects on TME and the effectiveness of radiation therapy in the regulation of tumor-related immune responses, angiogenesis, activation of CAFs in the tumor stroma, generation, and composition of tumor-secreted exosomes. The substantial amount of data indicates that radiotherapy impacts major components of TME, resulting in the activation of dual pro- and anti-inflammatory effects.

To improve overall survival and tumor eradication, radiotherapy may need to be combined with ICIs to target TME in some cancer patients. A combined approach may also prevent the development of radiotherapy resistance caused by the anti-inflammatory TME components. The strategy to use complex treatment protocols (radiotherapy combined with targeted anti-tumor drugs, including ICIs) has been successfully implemented in clinical research, although not all promising drugs have been tested using the combined protocol (Table 1). Recently obtained clinical trial data shows that combined therapies have substantial treatment advantages, although further testing is required.
